# Modulating iron metabolism and gut microbiota: the therapeutic potential of Chia Seed Oil in obesity-related diabetes

**DOI:** 10.3389/fmicb.2025.1676971

**Published:** 2026-01-28

**Authors:** Wencong Li, Li Zhang, Xiangxiang Wang

**Affiliations:** 1 Department of Endocrinology, Shandong Provincial Third Hospital, Shandong University, Jinan, Shandong, China; 2 Department of Internet Hospital, Shandong Provincial Third Hospital, Shangdong University, Jinan, Shandong, China

**Keywords:** obesity-related diabetes, iron metabolism, gut microbiota, Chia Seed Oil, microbiology

## Abstract

**Introduction:**

Obesity-related diabetes is a significant global health concern, underscored by perturbations in iron metabolism and gut microbiota composition. This study investigates the mechanistic role of Chia Seed Oil (CSO), rich in omega-3 fatty acids, in suppressing iron metabolism pathologies and promoting gut microbiota alterations to mitigate obesity-related diabetes.

**Methods:**

Using a high-fat diet-induced obesity model in male C57BL/6J mice, we aimed to explore the effects of CSO supplementation on metabolic outcomes, iron status, and gut microbiota diversity.

**Results:**

Our findings suggest that CSO effectively regulates iron metabolism, evidenced by altered serum ferritin levels, hepcidin, and transferrin saturation, while promoting a diverse gut microbiota profile.

**Discussion:**

The study elucidates the potential of CSO as a therapeutic agent in managing obesity-associated metabolic disorders by restoring iron homeostasis and fostering gut health. These results highlight the interconnectedness of dietary fat, iron metabolism, and microbiome dynamics in the pathophysiology of obesity-related diabetes, suggesting a multifaceted approach to treatment strategies.

## 1 Introduction

The worldwide increase in obesity constitutes a significant public health challenge of the 21st century, acting as a principal catalyst for the concurrent epidemic of Type 2 Diabetes (T2D) (Chu et al., 2022; Li et al., 2019). The essential connection between these two disorders is well-documented, focusing on a pathological cycle of energy surplus, adipose tissue proliferation, and persistent low-grade inflammation. This inflammatory condition, intensified by lipotoxicity due to ectopic fat accumulation in the liver and muscle, leads to insulin resistance, a characteristic of T2D (Su et al., 2023). As a result, pancreatic β-cells experience compensatory hyperfunction, ultimately resulting in fatigue and apoptosis, which leads to overt hyperglycemia. This paradigm of inflammation and insulin resistance is essential, although it provides an imperfect understanding of the underlying cellular diseases. Recent research has started to clarify the involvement of intricate mechanistic processes, such as dysregulated iron metabolism and a newly identified form of regulated cell death termed ferroptosis, in the development of metabolic disease (Jiang et al., 2021; Zheng and Conrad, 2020).

Ferroptosis is an iron-dependent, non-apoptotic form of cell death marked by the detrimental accumulation of lipid peroxides, resulting in the rupture of the plasma membrane. This mechanism is visually and biochemically unique from other forms of cell death (Mao et al., 2024). The execution relies on the depletion of glutathione peroxidase 4 (GPX4), the principal enzyme that neutralizes lipid hydroperoxides in cell membranes. The trigger for this deleterious sequence is the presence of catalytic ferrous iron (Fe^2+^). Obesity is increasingly acknowledged as a condition of iron overload, or dysmetabolic iron overload syndrome (Moore Heslin et al., 2021). This illness is caused by inflammation of adipose tissue, which disrupts the function of the iron-regulatory hormone hepcidin. Increased hepcidin sequesters iron among enterocytes and macrophages, resulting in elevated iron storage (indicated by high serum ferritin) and subsequent tissue iron accumulation (Ganz, 2005). The surplus labile iron accelerates the Fenton reaction, producing substantial reactive oxygen species that initiate lipid peroxidation chains, thus fostering conditions conducive to ferroptosis. The susceptibility of insulin-secreting pancreatic β-cells and insulin-sensitive tissues to oxidative stress renders ferroptosis a likely and important factor in the advancement of diabetes (Eguchi et al., 2021).

The gut microbiota is a crucial regulator of systemic iron homeostasis and metabolic health (Wang X. et al., 2025; Guo et al., 2025; Wang et al., 2010). Research indicates that a high-fat diet (HFD) causes gut dysbiosis, undermining intestinal barrier integrity and enabling the translocation of bacterial endotoxins such as lipopolysaccharide (LPS) into the bloodstream, hence exacerbating systemic inflammation (Cheng et al., 2022; Jiang et al., 2025). This dysbiosis is also associated with the dysregulation of iron absorption and storage. A high-fat diet can launch a detrimental cycle: food-induced dysbiosis fosters inflammation and iron overload, which then accelerates lipid peroxidation and ferroptosis, ultimately worsening metabolic dysfunction (Malesza et al., 2021). Focusing on the “gut microbiota-iron-ferroptosis axis” offers an innovative treatment approach to disrupt this loop (Gu et al., 2023; Wang Z. et al., 2025).

In this setting, natural therapies with pleiotropic effects present considerable potential (Chen et al., 2023; Li et al., 2024). Chia Seed Oil (CSO), extracted from *Salvia hispanica* L., is abundant in omega-3 polyunsaturated fatty acids, especially α-linolenic acid, and exhibits significant antioxidant capabilities (Motyka et al., 2022; Khalid et al., 2023). Prior research has shown its effectiveness in enhancing lipid profiles, decreasing oxidative stress, and alleviating insulin resistance in high-fat diet animals. Nonetheless, the current literature has predominantly ascribed these advantages to the overall antioxidant and anti-inflammatory properties of CSO. A considerable and unresolved information gap persists about its precise impact on the interrelated pathways of iron metabolism and ferroptosis. The relationship between CSO’s protective benefits and its capacity to alter gut microbiota, which may normalize systemic iron homeostasis and enhance cellular defenses against ferroptosis by maintaining GPX4 function, remains uncertain (Zeng et al., 2025; Safari et al., 2025).

This study was conducted to evaluate the new idea that CSO mitigates HFD-induced metabolic dysfunction by selectively targeting the gut microbiota-iron-ferroptosis axis. We propose that CSO supplementation reorganizes the gut microbial ecology, which mitigates inflammation and rectifies iron overload, thus diminishing the principal catalyst of lipid peroxidation. Simultaneously, we assert that CSO directly or indirectly enhances GPX4 defense mechanisms, protecting cells against ferroptotic death. To evaluate this, we examined the impact of CSO on critical indicators of this axis, encompassing gut microbiota beta-diversity, systemic iron status (serum ferritin), gene expression of iron storage [Ferritin Heavy Chain 1 (FTH1)] and anti-ferroptotic (GPX4) proteins, as well as the definitive marker of ferroptotic stress—lipid peroxidation. Our findings present compelling evidence for a novel mechanism behind CSO’s therapeutic potential, transcending a traditional antioxidant rationale to a complex, multi-system mode of action.

## 2 Materials and methods

### 2.1 Samples

Chia seeds were obtained from reputable domestic suppliers in China. The seeds were harvested and stored in vacuum packaging at −18 °C. A blender was used to ground the seeds and obtain the flour and prepare the experimental diet. The samples were stored in a black polypropylene bag at −18 °C to prevent lipid oxidation. The fatty acid composition of CSO was identified by gas chromatography mass spectrometry (GC–MS, make: Agilent, model: 6890-5973N).

### 2.2 Animals and housing

The animal studies were conducted on 30 male C57BL/6J mice (3 weeks) obtained from the Shandong University animal house. The animals were randomly divided into 5 experimental groups [(e.g., NC, HFD, HFD + CSO, HFD + 2-AB, HFD + 2-AB + CSO)], with 6 animals per group.

The study approved by the Ethics Committee of the Shandong University and was performed according to the Directive 86/609/EEC of November 24, 1986, in compliance with the ethical principles for animal experimentation. The animals were kept in individual polypropylene cages under controlled conditions [light/dark cycle (12 h) and temperature (22 °C ± 2 °C)] deionized water and diets ad libitum. The animals were accustomed for 1 week before the initiation of the experiment. The animals received a standard diet (STD comprised of 64% carbohydrate, 19% protein, and 17% fat) or a high-fat diet [HFD, comprised of 20.5% protein, 35.8% fat, 0.4% fiber, 3.6% ash, 3.1% moisture, and 36.8% carbohydrate (primarily disaccharides)].

### 2.3 Experimental design

Following a (one-week) acclimatization period, animals were randomly assigned to the different dietary groups based on (e.g., their initial body weight) to ensure equivalent starting points across all groups. Randomization was performed using a random number table. The animals were randomly divided into 5 groups (6 animals in each group): (1) Control group: Mice receiving the STD without any treatment. (2) HFD group: Mice receiving the HFD without any treatment. (3) HFD + CSO Group: Mice receiving the HFD and treated with CSO (1900 mg kg^−1^ BW). (4) HFD + 2-AB Group: Mice receiving the HFD and 2 antibiotics 2-AB, Dissolve enrofloxacin (0.575 mg/mL) and ampicillin (1 mg/mL). (5) HFD + 2-AB + CSO Group: Mice receiving the HFD and 2-AB and treated with CSO (1900 mg kg^−1^ BW). The 2-AB was administered via drinking water to the relevant groups (HFD + 2-AB Group and HFD + 2-AB + CSO Group) 2 weeks before initiating the HFD regimen and continued for the initial 4 weeks of the HFD regimen to achieve significant depletion of the gut microbiota. The dose of CSO was 1900 mg/kg body weight. This dosage was selected based on the study by Han et al. (2020), which demonstrated its efficacy in mitigating high-fat diet-induced metabolic disturbances and oxidative stress in mice. CSO was administered by gavage at week 17 after the induction of obesity-related diabetes by the HFD and continued for 18 weeks to assess both short-term and long-term therapeutic effects. The body weight and food intake were monitored weekly during the experiment. Throughout the experiment, the investigators involved in [e.g., the daily monitoring of animals and diet preparation] were not blinded due to the nature of the interventions. However, blinding was implemented during the outcome assessment and data analysis phase. Specifically, the personnel performing the [e.g., biochemical assays, RNA/protein expression analyses, histological scoring, and statistical analysis] were unaware of the group allocations.

At the end of the experiments, cardiac puncture was conducted to euthanize the animals after anesthesia with isoflurane (3%, Isoforine, Cristália®). The duodenum, cecal content, and cecum were collected, weighed and immediately stored at−80 °C for analysis.

To delineate the role of the gut microbiota in the observed metabolic effects, an antibiotic-treated group (2-AB) was included. This group served a dual purpose: (1) to determine if the therapeutic benefits of CSO were dependent on an intact gut microbiome, and (2) to create a model of microbiota depletion, allowing us to assess the contribution of dysbiosis to iron regulation and ferroptosis independently of the HFD. The combination group (HFD + 2-AB + CSO) was used to investigate potential interactions between CSO and a compromised microbial community.

### 2.4 Monitoring and measurements

#### 2.4.1 Blood glucose level

The blood glucose level was measured weekly using a glucometer (OneTouch Ultra2 glucometer, LifeScan, Inc.). The tail tip of animals was cut to release blood, which was applied to a test strip in a glucometer. Monthly blood glucose level was measured using Sigma assay kit (Cat. # GAHK20). The blood sample was obtained from the retro-orbital sinus under isoflurane anesthesia. The obtained sample was collected, centrifuged at 352 *g* for 15 min at 4 °C, and the resultant serum stored at −20 °C for measurement and analysis.

#### 2.4.2 Plasma insulin and lipid profile measurement

At the end of the experiment, the Ethylenediaminetetraacetic acid (EDTA)-nized blood samples were collected, centrifuged at 352 *g* for 15 min at 4 °C, and the resultant plasma was used to measure plasma triglycerides, total plasma cholesterol, HDL-cholesterol concentrations, and insulin level using commercial kits (Nanjing Jiancheng Biology Engineering Institute, Nanjing, China).

#### 2.4.3 Iron metabolism evaluation

Iron metabolism was evaluated by measuring the total iron-binding capacity (TIBC), iron concentration, ferritin level, transferrin level, serum transferrin saturation index (TSI), and hepcidin level. Chemiluminescence immunoassay (Beckman Coulter, United States) was used to measure Serum ferritin levels. The ferrozine colorimetric method (serum iron detection kit, Beckman Coulter, United States) was used to measure the serum iron levels. Unsaturated transferrin binding capacity (UIBC) levels was measured using a commercial kit (Beckman Coulter, United States) and applied to calculate TIBC (serum iron + serum UIBC). Serum hepcidin levels were quantified using the ELISA method.

The following equation was applied to calculate the serum transferrin saturation index (TSI):


$$\mathrm{TSI}\;(\%)=(\frac{\text{Serum iron}}{\text{TIBC}})\times 100$$

### 2.5 Evaluation of ferroptosis markers

The ferroptosis state under treatment with CSO was evaluated by measuring lipid peroxidation level, glutathione (GSH) levels, GPX4 activity, and iron accumulation state. The lipid peroxidation was evaluated by measuring malondialdehyde (MDA) level using commercial MDA assay kit (Nalondi™-Lipid Peroxidation Assay Kit-MDA). MDA is an indicator of the lipid peroxidation in biological and food samples. The GSH levels were evaluated using the enzymatic recycling method according to the previous method with slight modification (Shaik and Mehvar, 2006). Increased Gpx4 activity should result in improved defense against ferroptosis since Gpx4 is essential for preventing ferroptosis by lowering PLOOHs in the membrane. The Gpx4 activities were measured according to previous study with negligible alteration (Chen et al., 2021). The iron accumulation state in the tissues were evaluated using the Prussian blue staining. The idea behind Prussian blue staining is that iron and potassium ferrocyanide combine to create a compound that appears blue–black under a microscope. Iron deposits in tissues can be located using this reaction, which is exclusive to ferric iron (Fe^3+^).

The molecular studies on the ferroptosis pathways were conducted using gene expression (FTH1, FTL, and TFRC) and protein expression evaluations using quantitative PCR (qPCR) and western blotting, respectively. RNAiso Plus (TaKaRa, Japan) was used to isolate RNA from tissues. PrimeScript™ RT Master Mix (Takara, Japan) was used to synthesis cDNS form the isolated RNA (1 μg). The 7500 Real-Time PCR System (Thermo Fisher Scientific, USA) was used to conduct the PCR based on SYBR Green fluorescence (TaKaRa, Japan). The relative mRNA expression levels were calculated using the 2^−ΔΔCt^ method and normalized to the geometric mean of the reference gene, Actb, which was validated to be stable under our experimental conditions. Each sample was run in technical duplicate.

The lysis buffer (RIPA, Beyotime, China) was used to lyse the tissues. The BCA assay kit (Beyotime, China) was used to measure the protein concentrations. 10% sodium dodecyl sulfate-polyacrylamide gel (SDS-PAGE) was loaded with equal amounts of protein, which were subsequently moved from the gel to polyvinylidene fluoride membranes (Millipore, USA). The membranes were blocked for 1 h in a 5% bovine serum albumin (BSA, Solarbio, China) solution, followed by a TBST wash and an overnight incubation with primary antibodies at 4 °C. The antibodies listed below were employed:

The membranes were cleaned and then treated for 1 h at 37 °C with secondary antibodies conjugated with horseradish peroxidase (HRP): goat anti-rabbit IgG antibody (1:2000, #7074, Cell Signaling Technology, USA). The ECL western blotting detection system (Millipore, USA) was used to find bound antibodies.

### 2.6 Inflammatory and oxidative stress markers

The inflammation and oxidative stress sates of serum under treatment with CSO were evaluated by measuring serum levels of Tumor Necrosis Factor-alpha (TNF-α), IL-6 (Interleukin-6), and Reactive Oxygen Species (ROS). TNF-α and IL-6 levels were measured by ELISA technique using commercial kit (BioSource Europe SA, 8 B-1400, Nivelles, Belgium), according to the manufacture’s protocol. A commercial Reactive Oxygen Species Assay Kit (S0033, Beyotime, China) was used to measure the production of ROS.

### 2.7 Gut microbiota composition evaluation

The gut microbiota composition was evaluated using analysis of the fecal samples using 16S rRNA gene sequencing. A popular method for assessing the composition of the gut microbiota is 16S rRNA gene sequencing, which makes it possible to identify and describe the bacterial populations there. This technique determines the diversity and relative abundance of various bacterial species and genera by analyzing the 16S rRNA gene, a conserved gene found in all bacteria. The luminal content of the cecum was collected, divided for microbiome and meta-metabolome analyses, snap-frozen in liquid nitrogen, and stored at −80 °C. Liver samples were also collected for metabolomics analyses.

#### 2.7.1 DNA extraction and sequencing

Cecal content samples were mechanically disrupted and DNA extraction was conducted on 30 mg of cecal stool samples using the NucleoSpin for Soil Kit (Macherey-Nagel, Dueren, Germany), following the manufacturer’s instructions. The V6–V9 region of the 16S-rRNA gene was amplified and sequenced on a 454 GS FLX Titanium system (Roche). Sequences was processed and analyzed using other v.1.29.0,. Chimeric sequences were detected and removed using UCHIME. Using SILVA database v.138.1, the taxonomic classification and alignment of the sequences with the 16S rRNA gene were conducted. Sequences with ≥97% similarity were clustered into operational taxonomic units (OTUs). Good’s coverage estimator was used to evaluate each sample’s coverage (bacteria >97%). The smallest number of sequences generated from each sample was used to normalize the samples. The relative abundance of OTUs and alpha and beta diversity were computed using the normalized datatable. The alpha-diversity was estimated using the Chao1, Shannon, and Simpson indices. Principal Coordinate Analysis (PcoA), which was based on the Jaccard dissimilarity score, was used to evaluate the betadiversity between dietary groups. PICRUSt2 software was used to do a functional predictive study of the metagenome. Normalized OUT abundance was determined, and the Kyoto Encyclopedia of Genes and Genomes (KEGG) was used to predict the functional attributes that were allocated based on reference genomes. Plots were made of the most prevalent metabolic processes and the notable fold-change variations in functional pathways between the experimental groups. Additionally, the functional microbial pathways that were differently expressed in the experimental groups were identified using linear discriminant effect size analysis.

#### 2.7.2 Non-targeted metabolomics

The non-targeted Metabolomics were conducted on ceal and liver sample to identify detectable metabolites and metabolic pathways. The samples were homogenization in Tissue Lyser II (Qiagen) with cold methanol. Metabolomics analysis was performed using Fourier transform ion cyclotron resonance mass spectrometry (FT-ICR-MS, Bruker Daltonik GmbH, Germany) in both positive and negative ionization modes. The prepared samples were diluted in methanol in a ratio of 1–32 for stool samples and in 75% methanol in a ratio of 1–25 for liver samples prior to the MS experiment. Following that, the diluted samples were either sequentially infused at a steady 120 μL/h or put into a Gilson autosampler system. Cold condition (4 °C) was maintained on the autosampler’s well plate. For stool samples, 500 scans with an individual spectrum size of 2 MW and for liver samples, 400 scans with an individual spectrum size of 4 MW were used to acquire spectra in the range of 122.9–1000.0 m/z. The capillary and spray shield voltages were set to +3,600 V and +500 V, respectively, for the positive ionization mode, whereas the acquisitions in the negative ionization mode were set to −3,500 V and −500 V. Significant mass signals were identified and compared between groups using multivariate statistical analysis, including principal component analysis (PCA) and orthogonal partial least squares discriminant analysis (OPLS-DA).

### 2.8 Statistical analysis

In order to compare all experimental groups, data on murinometric variables were first subjected to a Kolmogorov–Smirnov normality test, followed by a one-way analysis of variance (ANOVA), and then, depending on the sample size, either Tukey’s *post hoc* test or the Tukey–Kramer post hoc test. Pearson’s correlation test was used to evaluate the relationships between inflammatory measures and gut health indicators. GraphPad Prism 9.0 was used for the analysis. The distance between the bacterial communities of animals from various dietary groups was displayed using a PCoA plot, which was based on the Jaccard dissimilarity metrics, to assess the clustering of the gut microbiome samples. The Past software was used to do a nonparametric analysis of similarities (PERMANOVA, number of permutations = 10,000). The Kolmogorov–Smirnov test was used to check for homogeneity of variance in datasets, and Dunn’s multiple comparison test was used to assess nonparametric and independent samples for the Kruskal–Wallis test. The STAMP software’s false discovery rate (FDR) was used to adjust the data for multiple comparisons. SPSS software version 20.0 with Bonferroni correction was used for statistical analysis. Statistical significance was determined at *p* < 0.05, and the data are displayed as means ± standard deviation.

All data presented in the study, including the qPCR results, biochemical assays (e.g., lipid peroxidation, serum ferritin), and Western blot analyses, were derived from biological replicates. Specifically, the sample size was *n* = 6 animals per experimental group. For qPCR and Western blot assays, each biological sample was run in technical duplicate to ensure measurement accuracy. The values presented as “means ± standard deviation” in the figures are based on these biological replicates (*n* per group).

## 3 Results and discussion

### 3.1 Effects of the diets on general health parameters

We evaluated the effects of the treatments on the animals’ cholesterol, triglycerides, cholesterol ester, blood glucose, glucose, and insulin levels.

**Figure 1:**
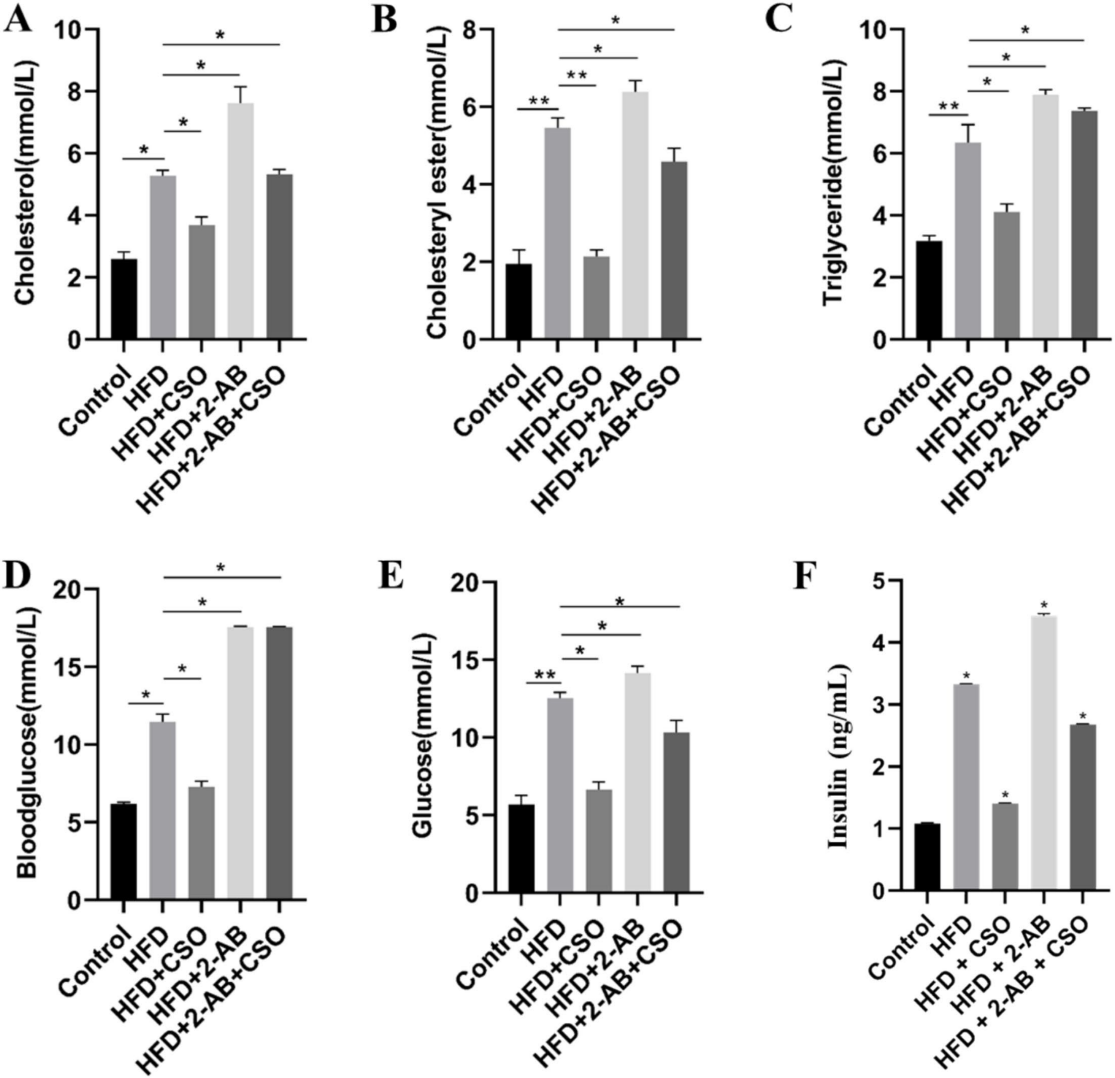
Effects of different diet consumption on **(A)** cholesterol, **(B)** cholesterol ester, **(C)** triglycerides, **(D)** blood glucose, **(E)** glucose, and **(F)** insulin level. Values represent means ± standard deviation. HFD, high fat die; CSO, Chia Seed Oil, 2-AB, Two Types of Antibiotics (enrofloxacin/ampicillin). (*) Indicates differences between the groups (paired test, *p* < 0.05).

We found that HFD consumption significantly (*p* < 0.05) increased the cholesterol level, while the consumption of HFD/CSO alleviated the effect of HFD (Figure 1A). Moreover, the effect of HFD/AB was greater than that of pure HFD, implying that microbiome depletion/stress using the AB predisposes the animals to hyperlipidemia under a high-fat diet. CSO treatment can alleviate the effect of HFD and has an indirect correlation with AB. We observed the same pattern on cholesterol ester (Figure 1B) and triglycerides (Figure 1C). The observed anti-hyperlipidemia of CSO can be related to its high content of alpha-linolenic acid (ALA), an omega-3 fatty acid.

Figure 1D shows that animals fed with HFD have higher BG than the animals consuming a standard diet, and modification of the diet with CSO alleviated the effect of HFD. The observed reduction of BG under treatment with CSO was significant and reduced the BG level around the NC group. On the other hand, CSO consumption could not alleviate the effect of HFD on BG and could not induce an antihyperglycemic effect on animals treated with AB. These observations indicate that the antihyperglycemic effect of CSO can be linked to the microbiota homeostasis. Figure 1E shows that consuming CSO can suppress the adverse effects of HFD and AB and reduce the glucose level. We measured the insulin level and found that HFD increased the insulin level, and CSO reduced the insulin level (Figure 1F). The adverse effect of HFD/AB on insulin level was more significant than pure HFD, and the HFD/CSO/AB consumption reduced the insulin level.

### 3.2 Effects of the diets on oxidative stress and inflammation

**Figure 2:**
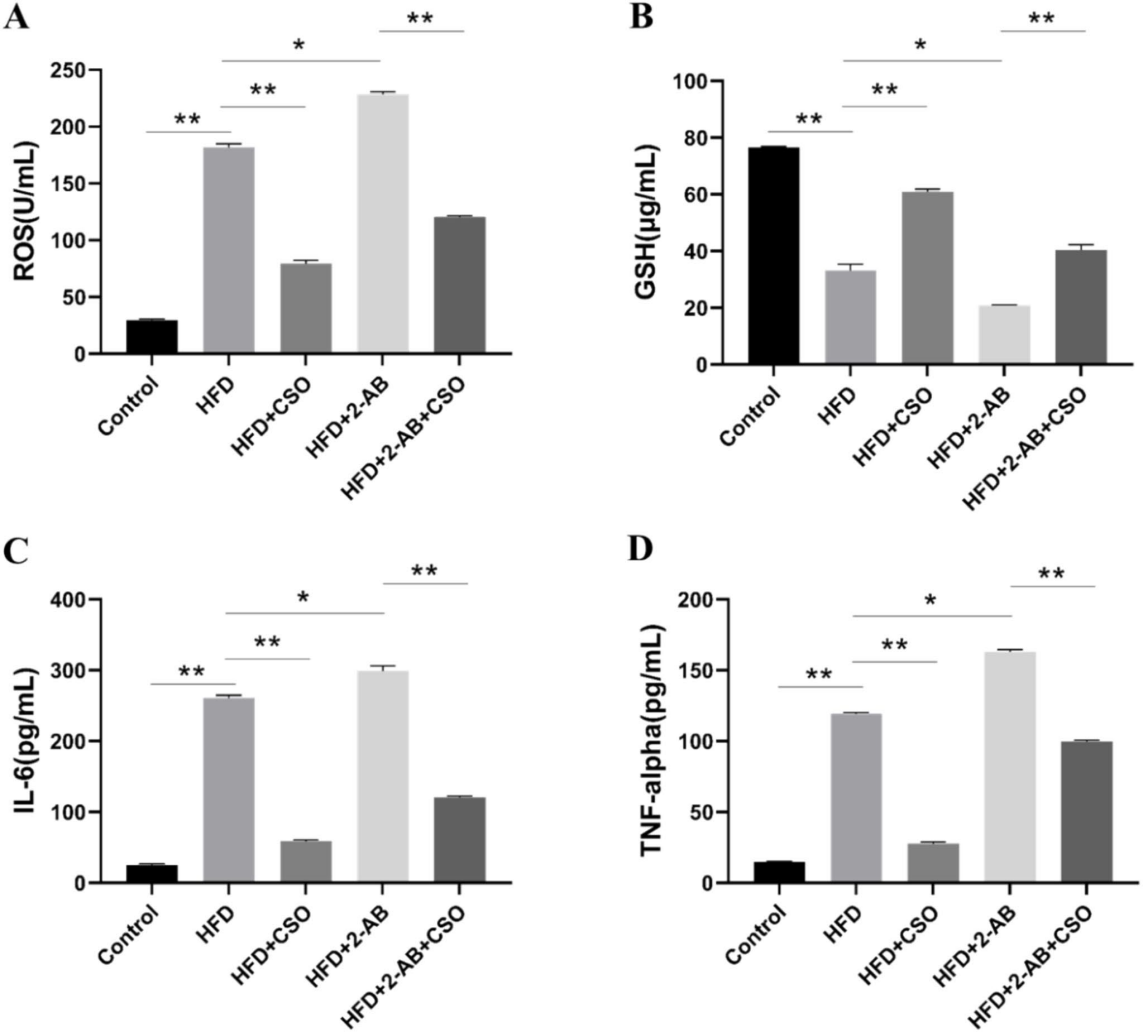
Oxidative stress and inflammation state of animals under different diets. **(A)** ROS level, **(B)** GSH level, **(C)** IL-6, and **(D)** TNF-α level. Values represent means ± standard deviation. HFD, high fat die; CSO, Chia Seed Oil; 2-AB, Two Types of Antibiotics (enrofloxacin ampicillin). (*) Indicates differences between the groups (paired test, *p* < 0.05). (**) Indicates differences between the groups (paired test, *p* < 0.01).

One of the leading causes of the onset and advancement of diabetes linked to obesity is oxidative stress, which is an imbalance between the body’s antioxidant defenses and the generation of reactive oxygen species (ROS). Elevated ROS, such as hydrogen peroxide and superoxide, can harm tissues and cells, resulting in several disorders linked to diabetes and obesity (Crujeiras et al., 2013). Increased adipose tissue, an endocrine organ that produces pro-inflammatory adipokines such as interleukin-1β (IL-1β), 6 (IL-6), and tumor necrosis factor-alpha (TNF-α), is a result of obesity. These chemicals promote the production of ROS, which exacerbates oxidative stress and low-grade chronic inflammation (Kang et al., 2016; Simons et al., 2005). Accordingly, we measured the ROS, GSH, IL-6, and TNF-α levels to evaluate the oxidative and inflammation states under different diets. We found that HFD consumption significantly (*p* < 0.001) increased the concentration of ROS, and its combination with 2-AB exacerbated the oxidative stress state (Figure 2A). On the other hand, CSO consumption reduced the ROS level and induced an antioxidant effect. The antioxidant activities of CSO can be due to its high content of antioxidants, like tocopherols, phenolic compounds, and phytosterols. Measuring the GSH level showed that HFD consumption significantly (*p* < 0.001) reduced the GSH level, and CSO intake modulated the GSH level (Figure 2B). Moreover, we found that HFD consumption significantly (p < 0.001) increased the IL-6 (Figure 2C) and TNF-α levels (Figure 2D), and CSO intake reduced the cytokines level and induced an anti-inflammatory effect. The observed anti-inflammatory effect of CSO can be related to its high content of omega-3 fatty acids, specifically ALA. It has been reported that ALA can regulate inflammation by influencing the production of eicosanoids (Zhu et al., 2020).

### 3.3 Effects of the diets on iron metabolism and ferroptosis

**Figure 3:**
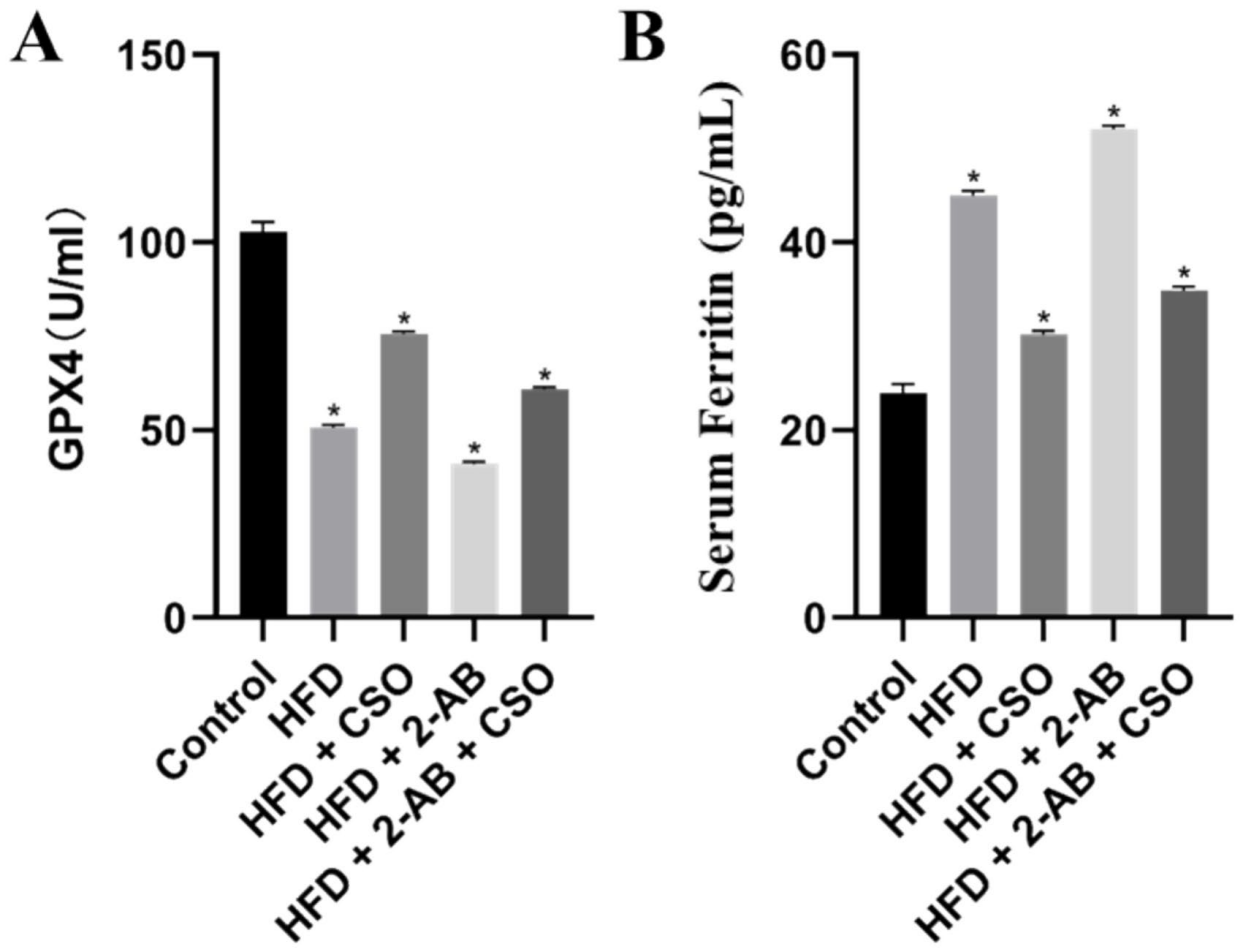
**(A)** GPX4 and **(B)** Serum Ferritin level under consumption of different diets. Values represent means ± standard deviation. HFD, high fat die; CSO, Chia Seed Oil; 2-AB, Two Types of Antibiotics (enrofloxacin ampicillin). (*) Indicates differences between the groups (paired test, *p* < 0.05).

Type 2 diabetes and obesity are linked to abnormalities in iron metabolism, which may affect several body processes. Increased hepcidin expression from obesity may decrease iron absorption and exacerbate iron insufficiency. On the other hand, a high body iron level has been connected to insulin resistance and fat storage, indicating that iron plays a part in developing diabetes and obesity (Fernández-Real et al., 2015). Research indicates a favorable correlation between body iron content, insulin resistance, and fat formation. While non-specific iron deficiency can result in anemia, iron decrease can help these disorders (Syrovatka et al., 2009; Vaquero et al., 2020). We measured the GPX4 level (Figure 3A) and serum ferritin level (Figure 3B) under different diets. We observed that HFD diet reduced the GPX4 level, and the combination of HFD with 2-AB resulted in the lowest GPX4 level.

On the other hand, CSO consumption elevated the GPX4 level and resulted in the highest GPX4 in the experimental groups. Maintaining a high level of GPX4 (an anti-lipo peroxidase) is critical to protect the cells (insulin-producing cell lines and primary islet cells) from ferroptosis (Krümmel et al., 2021; Deng et al., 2023). We also measured the Serum Ferritin level and observed that HFD consumption significantly (*p* < 0.05) increased the Serum Ferritin level, while the CSO diet modulated the Serum Ferritin level. It has been reported that there is a direct correlation between iron storage and obesity-related diabetes (Ikeda et al., 2022). Moreover, some studies reported that ferritin may play a role in insulin resistance and the development of type 2 diabetes (Khalil et al., 2018; Zhan et al., 2014). Our finding revealed that CSO consumption can regulate the HFD-induced abnormality of GPX4 and Serum Ferritin levels and protect the cells from ferroptosis.

**Figure 4:**
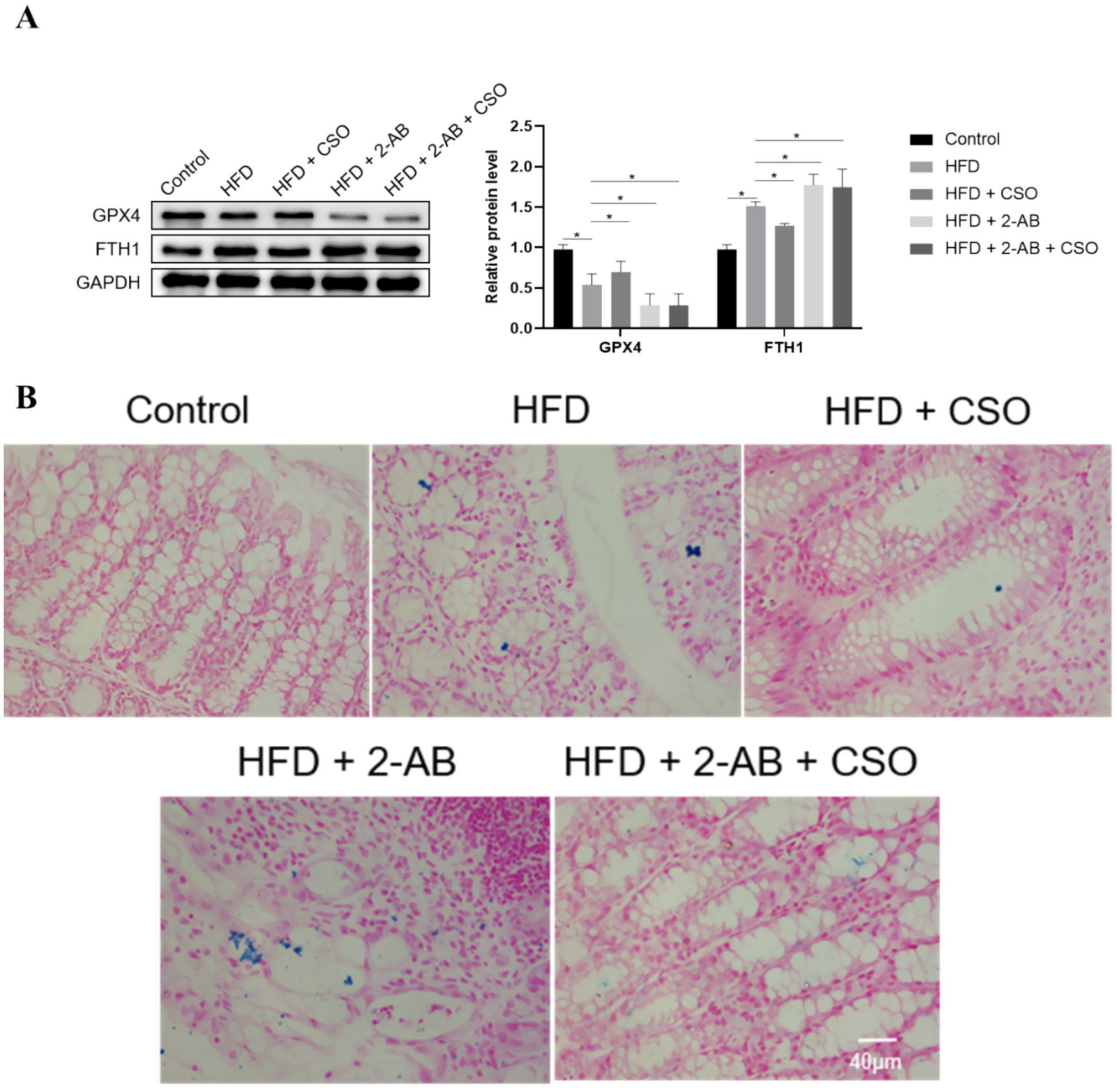
**(A)** Expression of GPX4 and FTH1 proteins in different groups and **(B)** deposition of iron in tissue in different groups, evaluated by prussian blue staining. Values represent means ± standard deviation. HFD: high fat die, CSO, Chia Seed Oil; 2-AB, Two Types of Antibiotics (enrofloxacin ampicillin). (*) Indicates differences between the groups (paired test, *p* < 0.05).

Notably, CSO treatment mitigated the effects of obesity-induced GPX4 expression (Figure 4A). In HFD-fed mice, CSO increased GPX4 expression while decreasing iron buildup and lipid peroxidation. These studies indicate that abnormal expression of GPX4 and FTH1 in adipose tissue is closely associated with obesity, and these are also essential regulators of ferroptosis. In conclusion, the previous results show that ferroptosis is closely associated with obesity, and it is suggested that targeting anti-ferroptosis may hold promise as a potential therapeutic strategy for obesity. The iron accumulation state in the tissues was evaluated using the Prussian blue staining. The idea behind Prussian blue staining is that iron and potassium ferrocyanide combine to create a compound that appears blue–black under a microscope. Iron deposits in tissues can be located using this reaction, exclusive to ferric iron (Fe3+). The results (Figure 4B) showed that the HFD consumption significantly increased iron accumulation, which was higher in animals fed with HFD + 2-AB. On the other hand, the iron deposition/accumulation was alleviated by consuming CSO.

**Figure 5:**
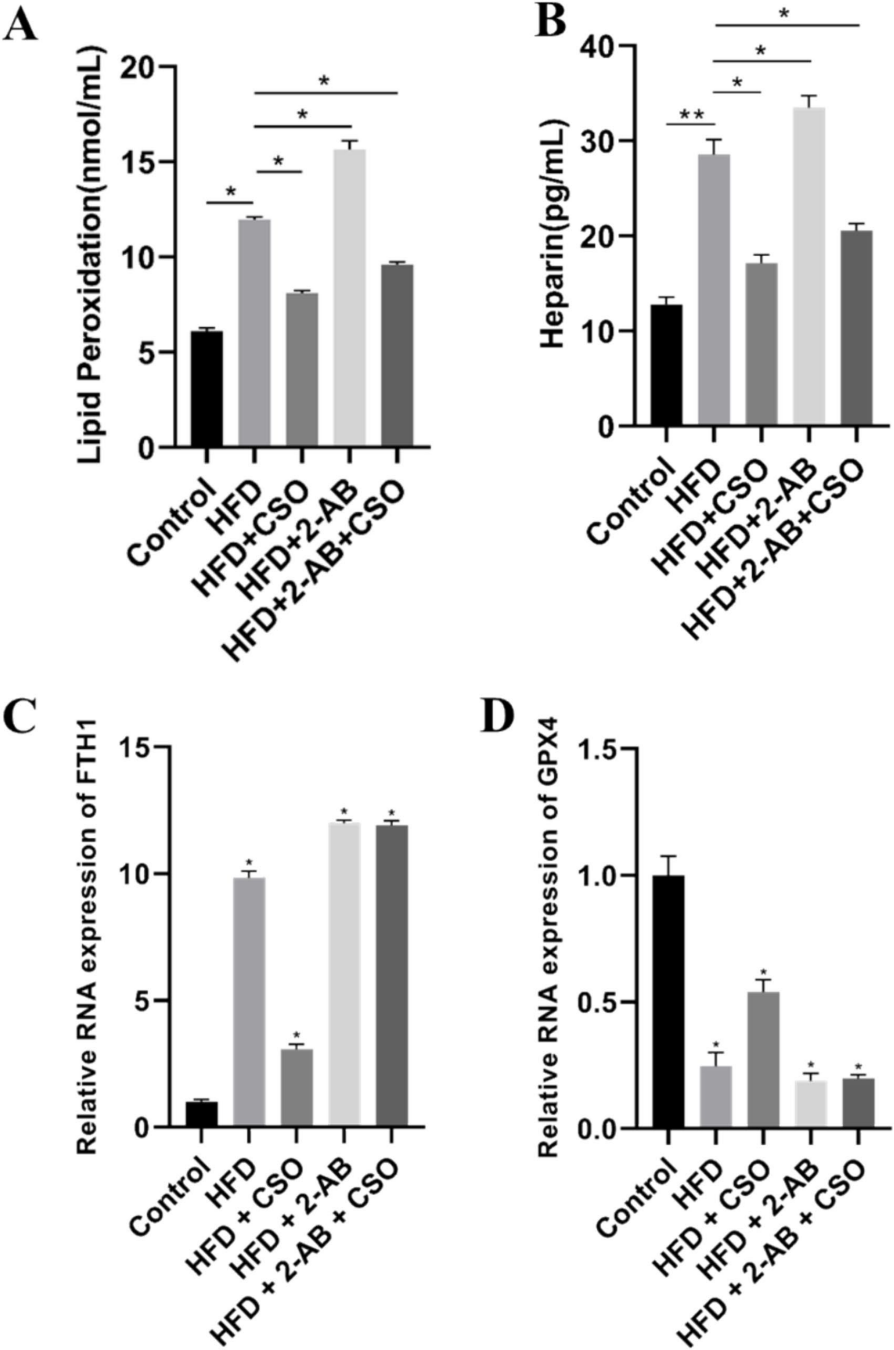
**(A)** Lipid peroxidation level, **(B)** heparin level, **(C)** FTH1 RNA expression, and **(D)** GPX4 RNA expression. Values represent means ± standard deviation. HFD, high fat die; CSO, Chia Seed Oil; 2-AB, Two Types of Antibiotics (enrofloxacin ampicillin). (*) Indicates differences between the groups (paired test, *p* < 0.05).

We evaluated the ferroptosis state underfeeding with different diets by measuring lipid peroxidation, heparin levels, and the relative expression of GPX4 and FTH1 mRNA. We found that HFD consumption significantly (*p* < 0.05) increased lipid peroxidation, and CSO consumption reduced it (Figure 5A). Interestingly, suppressing/shocking the microbiome through 2-AB consumption boosts lipid peroxidation, indicating the key role of the healthy microbiome in protecting cells from ferroptosis. We observed the same pattern of lipid peroxidation at the heparin level (Figure 5B). Heparin and ferroptosis are related because iron metabolism is regulated and affects this type of cell death. Heparin can change iron levels and the activity of proteins like DMT1, which is involved in iron uptake, by inhibiting hepcidin, a protein involved in iron control. This could have an impact on ferroptosis. This implies that ferroptosis may be affected if heparin is used to target iron homeostasis (Zhang et al., 2021). The results of the relative RNA expression of FTH1 (Figure 5C) and GPX4 (Figure 5D) were consistent with the results of the protein expression level.

### 3.4 CSO changes the intestinal microbiota pattern promoted by the HFD diet

**Figure 6:**
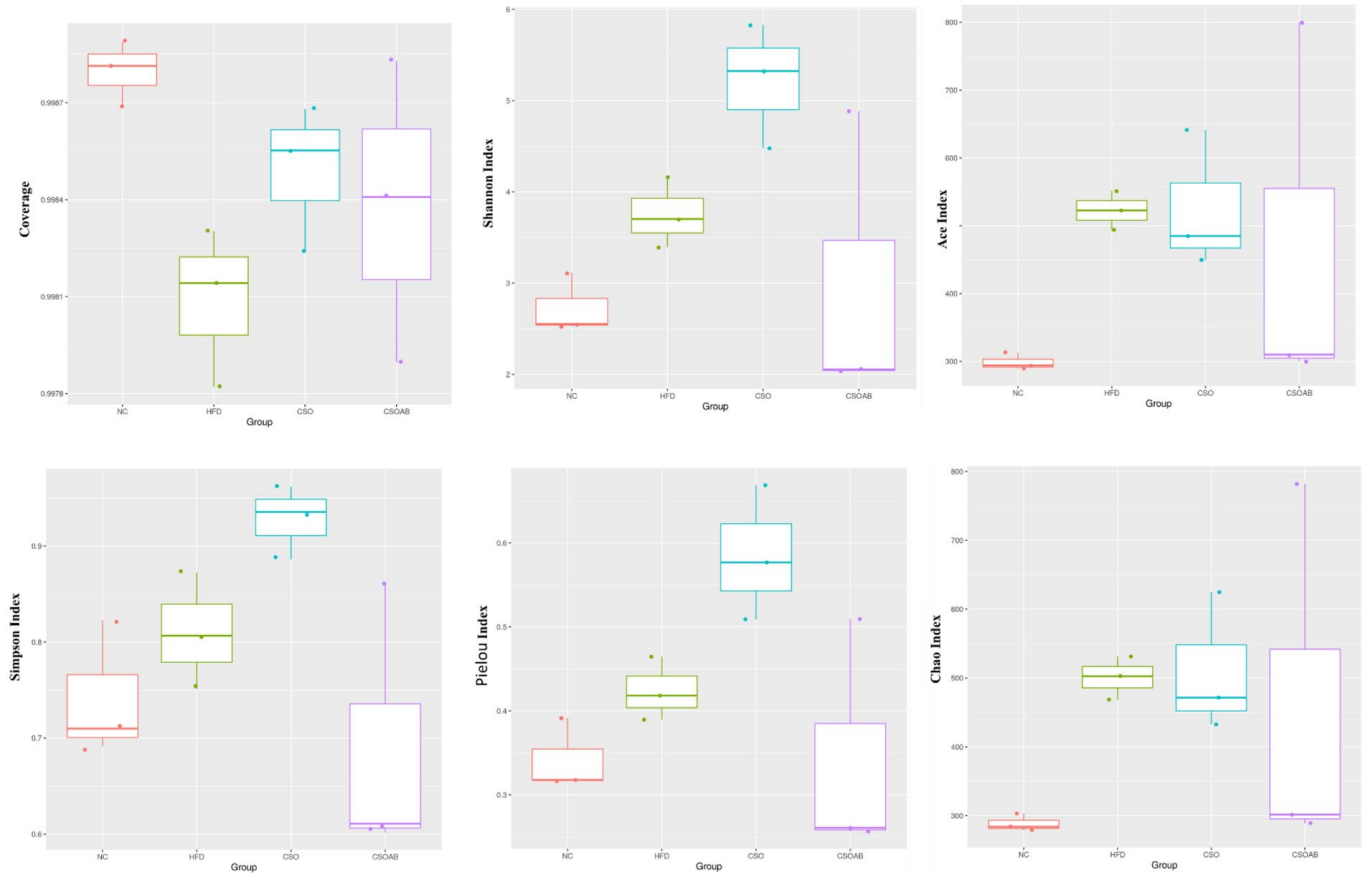
Alpha diversity metrics of bacterial communities in the cecal content of male C57BL/6J mice according to each treatment group. Data are expressed as median; bars = minimum and maximum values.

We evaluated the alpha diversity indexes of bacterial communities in the animals under different diets (standard diet, HFD, CSO, and SCO/AB) and the results are presented in Figure 6. We found that HFD increased the Shannon index, ACE index, Simpson index, Pielou index, and Chao index compared to the NC. CSO consumption induced the same effect and increased the Shannon index, ACE index, Simpson index, Pielou index, and Chao index compared to the NC. However, SCO/AB modulated the increased indexes and reduced Shannon index, ACE index, Simpson index, Pielou index, and Chao index compared to the NC HFD and CSO. These observations indicate that the consumption of CSO significantly increased the indicated indexes and implied that CSO consumption increased the richness of the bacterial community and the number of different species, as well as an increase in microbial diversity and how different the species are within the community. The indicated factors (richer and more diverse microbiota) revealed that CSO consumption improved the microbiota homeostasis and can be more resistant to external factors (Rinninella et al., 2019).

**Figure 7:**
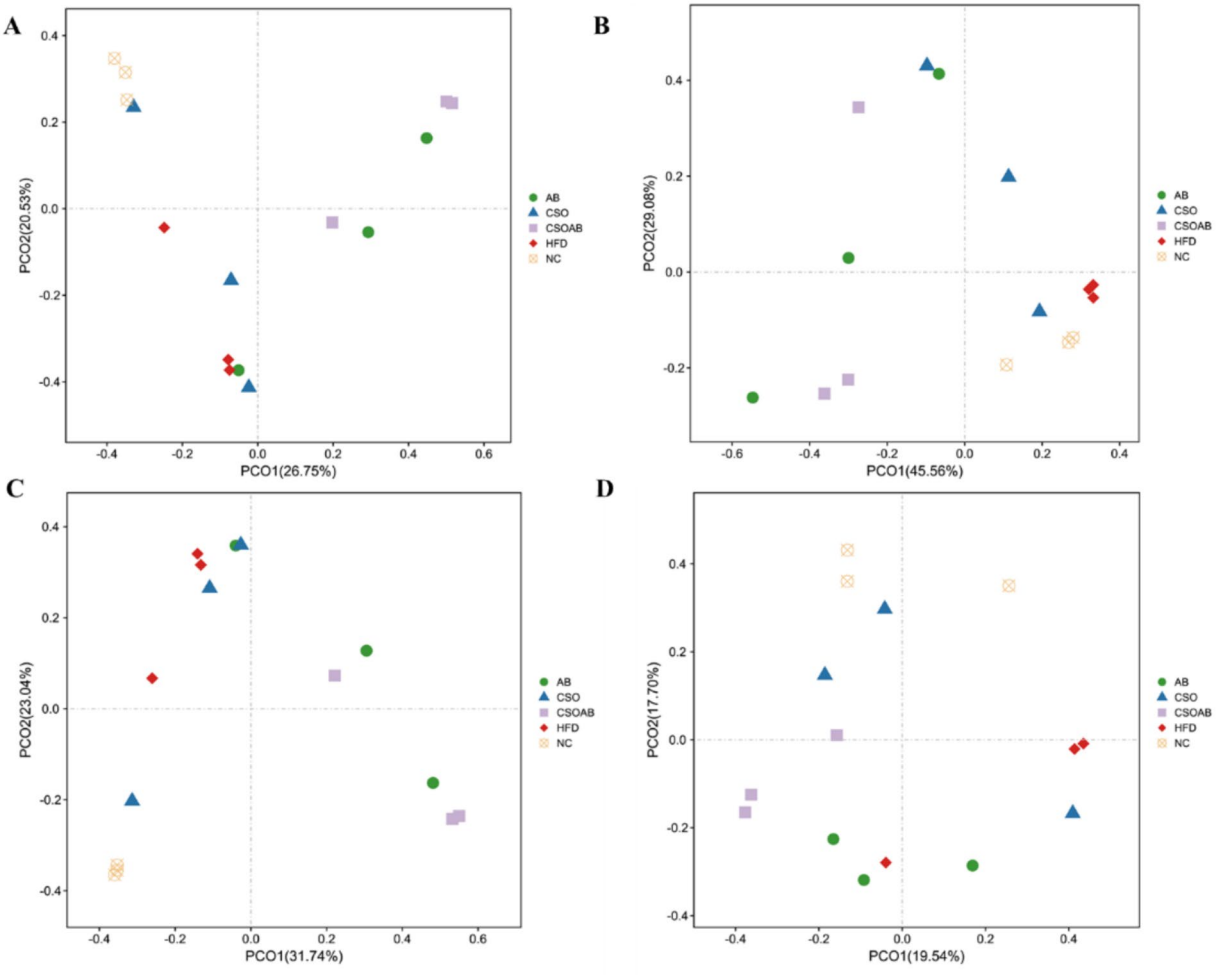
Changes in the β-diversity of the cecal content microbiome of male C57BL/6J mice according to each treatment group. **(A)** PCoA based on the Jaccard distance at the OTU level. **(B)** PCoA based on the Jaccard distance at the phylum level. **(C)** PCoA based on the Jaccard distance at the genus level, and **(D)** PCoA based on the Jaccard distance at the species level.

We conducted the beta diversity analyses of the cecal microbiome using Jaccard distances, and the results are presented in Figure 7. We observed that the consumption of the four types of diet-induced some variation in bacterial communities at the OTU, phyla genera, and Species level. The spatial ordination at the OTU level revealed that the animals fed with HFD were different from NC and CSO (Figure 7A). The evaluation at the phylum level showed that there was also a difference between the groups (Figure 7B). The evaluation at the genus level showed that the animals that received NC were clustered differently than HFD and CSO/NA (Figure 7C). At the species level, the HFD and NC clustered differently (Figure 7D). Beta diversity evaluations provide information about the degree of association or similarity between communities (Chao et al., 2019). We found that the animals fed with CSO cluttered differently, mainly on a HFD, revealing that their bacterial compositions were different.

### 3.5 CSO effect on the dominant cecal microbiota

**Figure 8:**
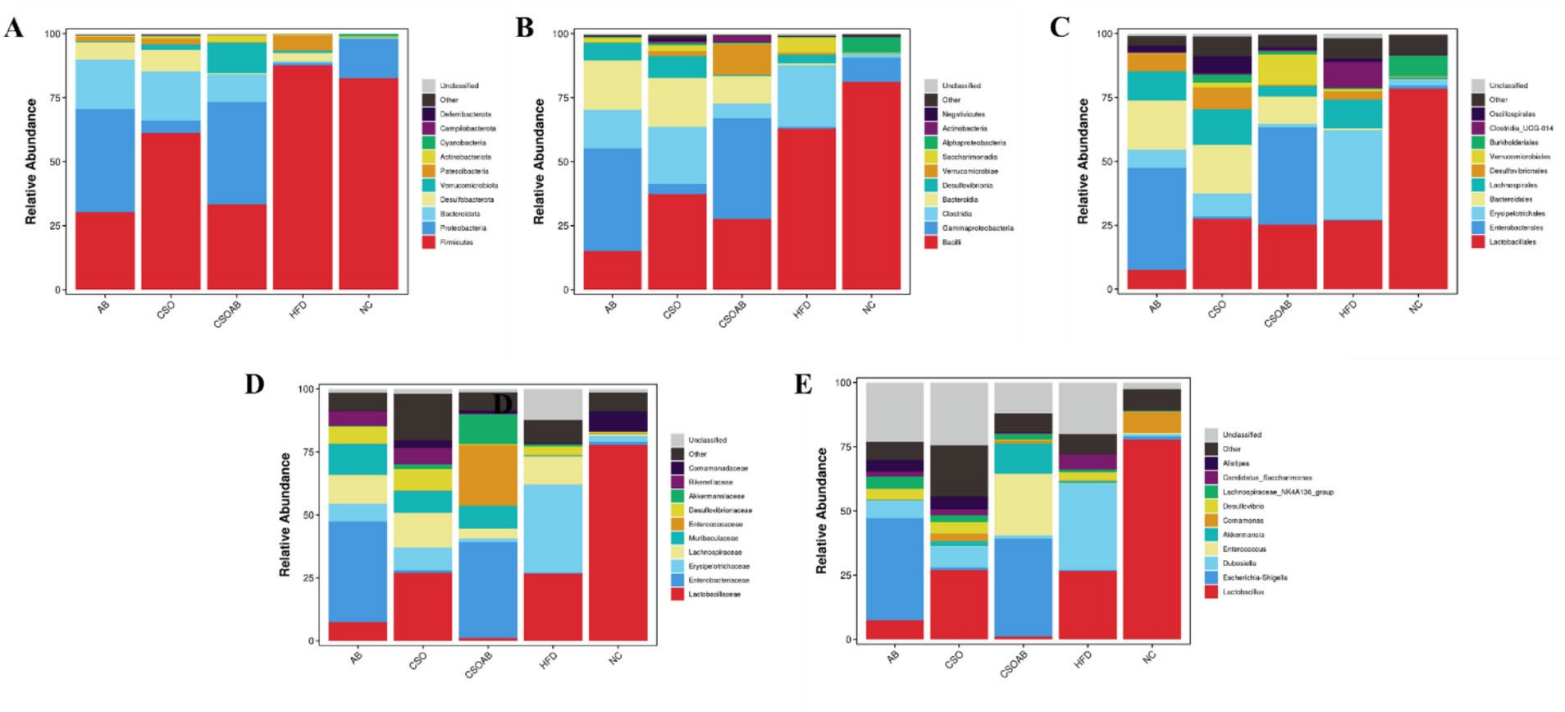
Dynamics of the relative abundance of bacterial taxa based on 16S rRNA sequencing at the phylum **(A)**, class **(B)**, order **(C)**, family **(D)**, and genus **(E)** levels in the cecal content of male C57BL/6J mice.

The stratification of class phylum, class, order, family, and genus is plotted in Figure 8. The results showed that although no difference was found in the relative abundance of the identified phyla between the NC and HFD, there was a difference between groups consuming a regular diet and HFD with AB, CSO, and AB/CSO. We found that Firmicutes, Bacteroidetes, and Proteobacteria were the main microorganisms in all groups. The same trend was observed at the class level (Figure 8B), but at the order level, the experimental groups are similar and different than NC (Figure 8C). The pattern for family (Figure 8D) and genus (Figure 8E) levels was the same, and the experimental groups were different from NC. At the class level, Bacilli and Gammaproteobacteria dominate all groups, but the population of Bacteroides was increased in AB, CSO, and CSO/AB groups. At the order level, Lactobacillales and Enterobacterales are dominant. At the family level, Lactobacillaceae and Enterobacteriaceae are dominant. At the genus level, *Lactobacillus* and *Escherichia-Shigella* are dominant.

**Figure 9:**
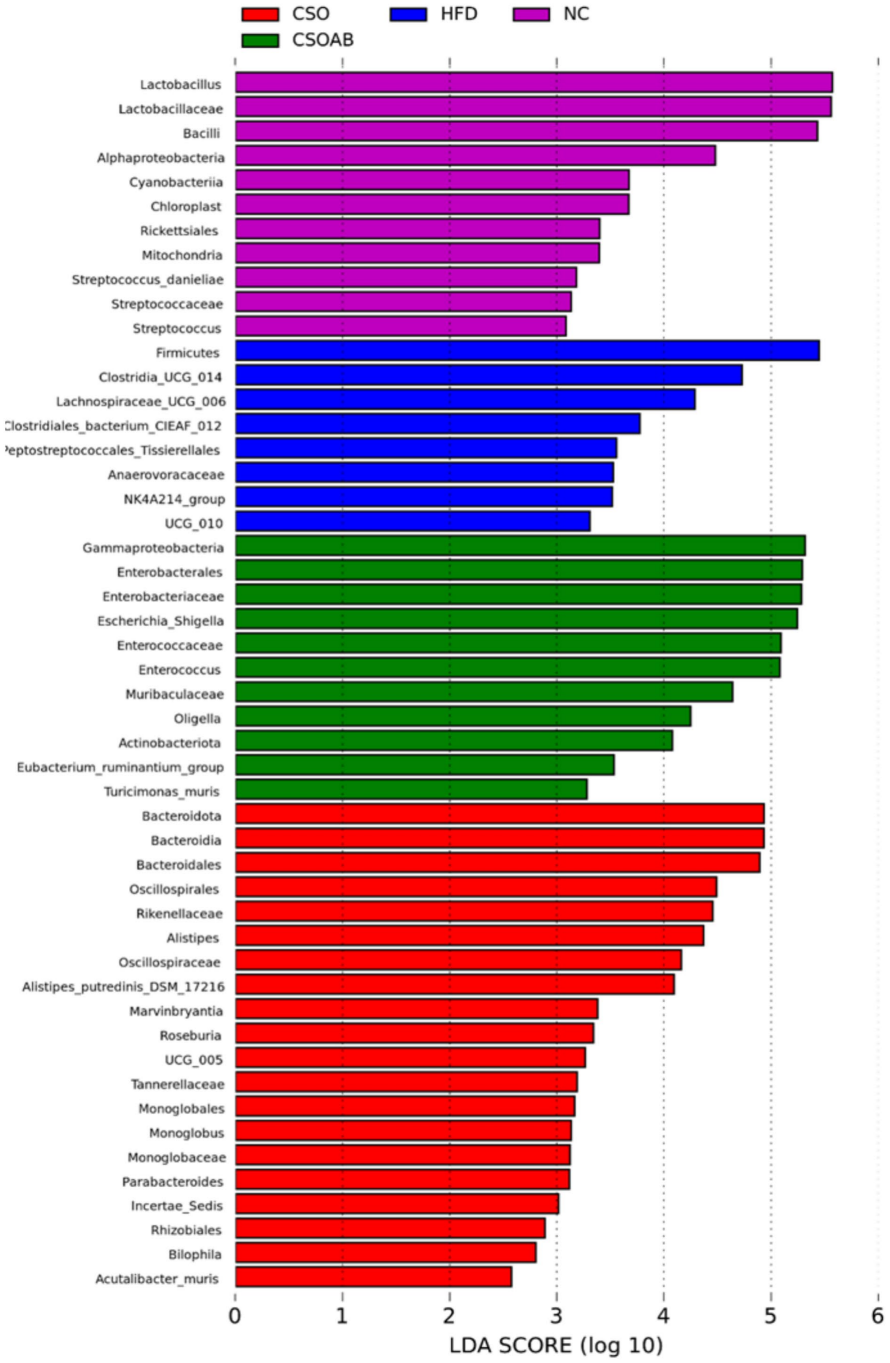
Histogram of linear discriminant analysis effect size (LEfSe) method to compute linear discriminant analysis (LDA) scores of differences in dominant microorganisms between groups.

The dominant cecal microbiota and intestinal biomarkers among the treatment groups were evaluated using the linear discriminant analysis effect size (LEfSe) method, and the results are presented in Figure 9. We found 24 dominant OTUs with an effect size of >4%. The CSO/AB group exhibited higher bacterial taxa than the control groups, with a larger Gammaproteobacteria and Enterobacteriaceae genera effect size. Lactobacillales and Lactobacillus are dominant in the NC group. We found that CSO consumption reduced the richness of pathogenic bacteria.

### 3.6 Proposed mechanistic model

**Figure 10:**
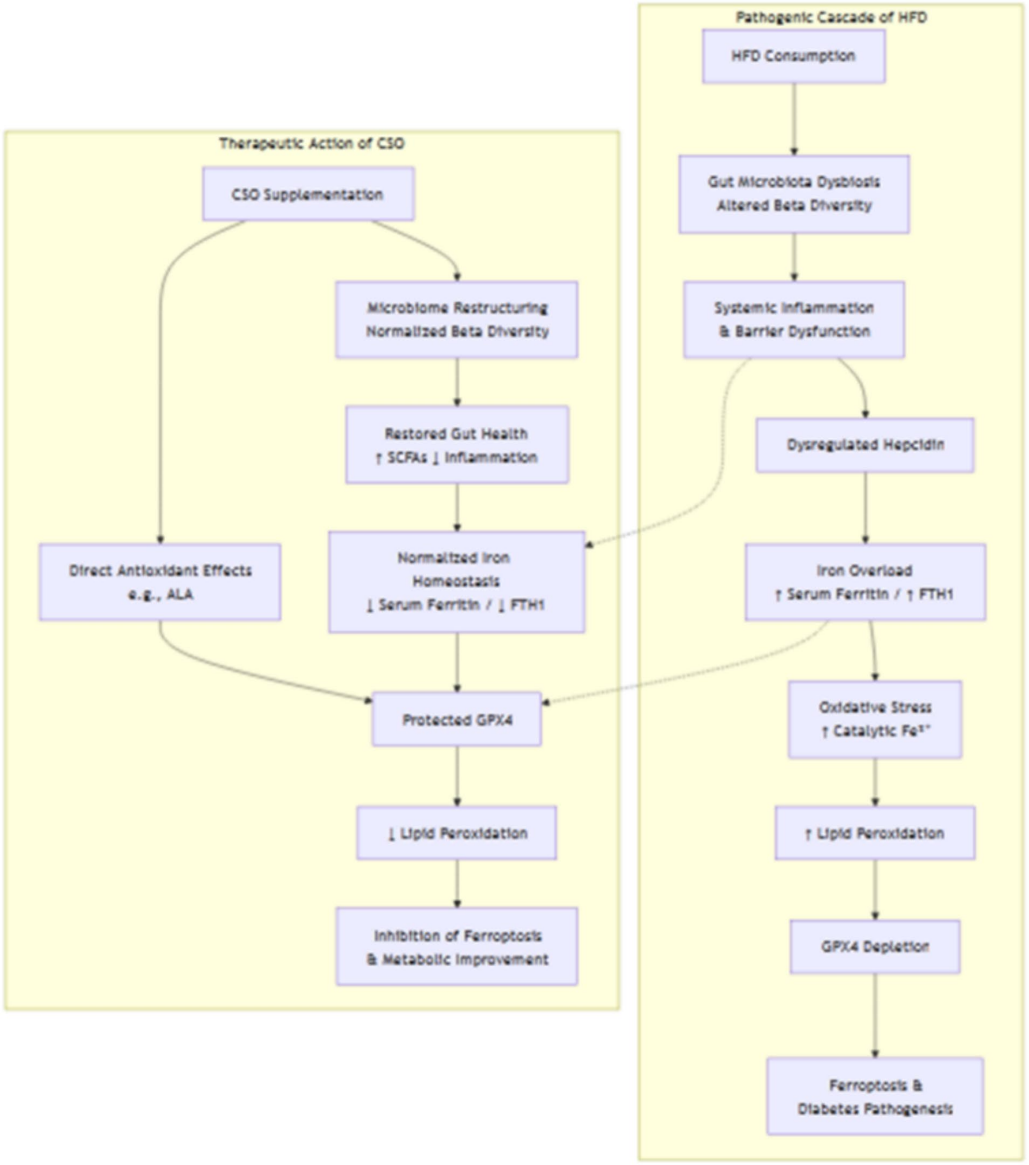
Proposed mechanistic model for the therapeutic action of Chia Seed Oil (CSO) in obesity-related diabetes.

Our results demonstrate that CSO mitigates obesity-related diabetes by modulating the gut microbiota–iron–ferroptosis axis (Figure 10).

A high-fat diet (HFD) disrupts gut microbial balance, as shown by altered beta diversity, triggering dysbiosis and disturbance of intestinal homeostasis. This dysbiosis promotes systemic inflammation and dysregulation of iron metabolism, reflected by elevated serum ferritin and FTH1 expression, indicating iron overload. Antibiotic-induced microbiota changes further confirm the gut microbiome’s central role in controlling host iron balance.

Iron overload subsequently enhances oxidative stress by increasing catalytic Fe^2+^ levels, which accelerate the Fenton reaction and intensify lipid peroxidation. This process reduces GPX4 expression at both protein and mRNA levels, promoting ferroptosis—a key driver of cellular damage in diabetes.

CSO disrupts this cascade through multiple mechanisms. At the microbiome level, it reshapes gut microbial composition, restoring balance and reducing inflammation-driven iron dysregulation. At the iron metabolism level, CSO normalizes ferritin and FTH1 expression, alleviating overload. Functionally, CSO suppresses ferroptosis by limiting lipid peroxidation, enhancing antioxidant defense, and preserving GPX4 activity.

Integrative analysis reveals a coherent mechanistic pathway:

HFD → Gut Dysbiosis → Systemic Inflammation → Iron Overload (↑Ferritin/FTH1) → Lipid Peroxidation → GPX4 Depletion → Ferroptosis → Diabetes Progression.

CSO Intervention → Gut Microbiota Reconfiguration + Antioxidant Action → Iron Homeostasis Restoration → ↓Lipid Peroxidation + ↑GPX4 → Ferroptosis Inhibition → Metabolic Protection.

The diagram depicts the detrimental cascade triggered by a HFD and the multifaceted response by CSO. Left (Red Arrow Pathway): High-fat diet consumption induces gut microbiota dysbiosis (demonstrated by modified beta diversity), resulting in systemic inflammation and the dysregulation of the iron regulator hepcidin. This leads to iron accumulation, marked by elevated serum ferritin and FTH1 expression. Excess catalytic iron (Fe^2+^) exacerbates oxidative stress through the Fenton reaction, resulting in increased lipid peroxidation. This overwhelms and depletes the essential antioxidant enzyme GPX4, ultimately inducing ferroptosis, a type of cell death that plays a role in the pathophysiology of diabetes. The CSO supplementation mitigates this cascade by (1) reorganizing the gut microbiome, as indicated by normalized beta diversity clustering, which diminishes inflammation and restores iron homeostasis, and (2) delivering direct antioxidant benefits. CSO effectively diminishes lipid peroxidation by normalizing iron levels and directly safeguarding GPX4, thereby reducing ferroptosis and enhancing metabolic function. Abbreviations: HFD (high-fat diet), CSO (Chia Seed Oil), FTH1 (Ferritin Heavy Chain 1), GPX4 (Glutathione Peroxidase 4), SCFAs (Short-Chain Fatty Acids).

The inclusion of the antibiotic-treated (2-AB) group was a crucial experimental design choice to move beyond correlation and probe the mechanistic role of the gut microbiota in our proposed “gut microbiota-iron-ferroptosis axis.” The purpose was threefold:

#### 3.6.1 To test for causal involvement, not just association

Observing that HFD alters the microbiome and that CSO improves outcomes is correlative. By depleting the microbiota with a broad-spectrum antibiotic cocktail (enrofloxacin and ampicillin), we created a model of *“microbiota-less”* or severely dysbiotic state.

*The key question was*: Are the benefits of CSO dependent on the presence of a gut microbiome? If CSO’s effects are *entirely mediated* by modulating the microbiota, then its protective effects should be *abolished or severely attenuated* in the antibiotic-treated groups (HFD + 2-AB and HFD + 2 - AB + CSO). If CSO has *direct, microbiome-independent effects* (e.g., systemic absorption of ALA providing direct antioxidant activity), then its benefits should *persist* even after antibiotic treatment.

#### 3.6.2 To isolate the microbiome’s role in iron regulation

The gut microbiota is a known master regulator of host iron homeostasis. Antibiotic-induced dysbiosis is a well-established model for disrupting this regulation (Das et al., 2020). By using the 2-AB group, we could directly test whether the HFD-induced iron overload (high serum ferritin/FTH1) is mediated by microbial changes. The findings in this group helped confirm that disrupting the microbiome directly impacts host iron storage.

#### 3.6.3 To investigate a novel synergistic interaction

The most intriguing result came from the *HFD+2-AB+CSO* group. We observed that CSO’s ability to reduce lipid peroxidation was not just maintained but was *potentiated* in the context of a depleted microbiome. This unexpected finding suggests a more complex interaction: CSO may not simply require a healthy microbiome, but may be particularly effective at *actively remodeling a disrupted microbial environment* or that its direct antioxidant effects are most crucial when the microbiome-derived oxidative stress is high. This provides a profound insight that would be impossible to gain without the antibiotic group.

In summary, the antibiotic group was not merely a control but a *critical mechanistic tool* to dissect causality, validate the microbiome’s role in iron metabolism, and uncover a novel, synergistic dimension of CSO’s action.

According to the results, we explicitly proposed and described the following interconnected mechanistic pathways to address this point:

1. The microbiota-iron-ferroptosis axis:

- We posited that a high-fat diet-induced dysbiosis undermines gut barrier integrity, potentially elevating systemic concentrations of microbial-derived chemicals such as lipopolysaccharide (LPS). This chronic inflammation can disrupt the primary iron regulator hepcidin, resulting in heightened iron absorption and accumulation in tissues (elevated serum ferritin levels).
- The excess iron facilitates ferroptosis by supplying ample catalytic iron (Fe^2+^) for lipid peroxidation. The subsequent decrease of GPX4 renders cells susceptible to this type of cell death.

1. The Protective Mechanism of CSO:

- We posited that CSO, abundant in α-linolenic acid (ALA) and fiber, functions at two critical junctures:

- Node 1: microbiota and iron homeostasis: we proposed that CSO supplementation promotes the proliferation of advantageous bacteria that generate short-chain fatty acids (SCFAs), which are recognized for enhancing the gut barrier and mitigating inflammation. The restoration of homeostasis can normalize hepcidin expression, therefore rectifying the dysregulated iron metabolism (reducing serum ferritin) identified in our study.
- Node 2: direct antioxidant and GPX4 assistance: we predicted that ALA from CSO can be integrated into cell membranes and/or act as a precursor for anti-inflammatory and antioxidant metabolites. This would diminish the total oxidative stress burden, hence preserving and potentially enhancing GPX4 activity. Moreover, by regulating iron concentrations (Node 1), CSO eliminates the principal catalyst for the Fenton reaction, so indirectly safeguarding GPX4 from excessive burden.

1. Synthesizing the pathway.

- We establish a more coherent narrative: CSO’s modification of the gut microbiota serves as the primary catalyst that facilitates the normalization of systemic iron levels. Rectifying iron metabolism subsequently diminishes the catalyst of lipid peroxidation, which collaborates synergistically with the prospective direct antioxidant properties of CSO to maintain GPX4 functionality and impede ferroptosis.

### 3.7 Limitations and future perspectives

Although our data presents persuasive evidence for a unique gut microbiota-iron-ferroptosis pathway in high-fat diet-induced metabolic dysfunction and its regulation by CSO, numerous limitations must be recognized. Initially, our sample size, although comparable to analogous pre-clinical investigations, was very limited; more research involving bigger cohorts is necessary to enhance the statistical power and generalizability of our findings. Secondly, whereas our data strongly indicate a mechanistic pathway, they do not furnish causal evidence. The creation of tissue-specific knock-out models (e.g., for GPX4 or FTH1) and fecal microbiota transplantation (FMT) experiments from CSO-treated donors to HFD-fed recipients will be crucial future measures to conclusively determine causality within this axis. The clinical translatability of our findings necessitates meticulous evaluation. The CSO dose administered (1900 mg/kg) corresponds to a Human Equivalent Dose (HED) of around 154 mg/kg, calculated by dividing the mouse dose by 12.3, which equates to roughly 10.8 g/day for a 70 kg human, utilizing body surface area normalization (Nair and Jacob, 2016; Jacob et al., 2022). Although this dietary intake is conceivable, the pharmacokinetics and overall efficacy of this dosage in humans have yet to be shown through clinical research. Notwithstanding these constraints, our research reveals a hitherto unidentified mechanistic network and establishes CSO as a promising natural product for further advancement in the treatment of metabolic disorders.

## 4 Conclusion

In the present study, we showed that CSO consumption alleviated the adverse effects of the HFD diet on lipid profile, blood glucose, oxidative stress, inflammation, ferroptosis, and microbiota of animals. The observed anti-inflammatory effect of CSO can be related to its high content of omega-3 fatty acids, specifically ALA. It has been reported that ALA can regulate inflammation by influencing the production of eicosanoids. Our finding revealed that CSO consumption can regulate the HFD-induced abnormality of GPX4 and Serum Ferritin levels and protect the cells from ferroptosis. The benefits of CSO on the microbiota can be more noticeable during an HFD. Consuming CSO, high in α-linolenic acid and insoluble dietary fiber, can improve the microbiota’s functionality by increasing the synthesis of advantageous metabolites. Additionally, our findings show that consuming chia flour increases SCFA synthesis while maintaining the relative abundance of the microbiota.

In conclusion, our data clarifies a new molecular pathway by which CSO mitigates obesity-related diabetes by interfering with the gut microbiota-iron-ferroptosis axis. The CSO-facilitated restoration of a balanced gut microbiome, along with its inherent antioxidant characteristics, regulates iron homeostasis and enhances cellular defenses against lipid peroxidation, thus protecting tissues against ferroptotic cell death. This complex mechanism highlights the considerable therapeutic potential of Chia Seed Oil as a natural treatment for metabolic disorders. While there are just a few studies assessing chia consumption and its effects on intestinal human health, more research is still required to fully understand how chia affects intestine health and microbiota and to make clearer what the potential ramifications for the human population may be. Our pre-clinical findings suggest that CSO may represent a promising nutraceutical candidate worthy of further investigation in the context of human metabolic disease.

## Data Availability

The data presented in this study are publicly available. The data can be found here: https://www.ncbi.nlm.nih.gov/bioproject/PRJNA1399618, BioProject Accession Number: PRJNA1399618; BioSample IDs: SAMN54501137 to SAMN54501151; SRA Accession Numbers: SRR36719129 to SRR36719143.
